# Oncolytic Vesicular Stomatitis Virus as a Viro-Immunotherapy: Defeating Cancer with a “Hammer” and “Anvil”

**DOI:** 10.3390/biomedicines5010008

**Published:** 2017-02-10

**Authors:** Michael Karl Melzer, Arturo Lopez-Martinez, Jennifer Altomonte

**Affiliations:** Klinik und Poliklinik für Innere Medizin II, Klinikum Rechts der Isar, Technical University of Munich, Ismaninger Str. 22, 81675 Munich, Germany; michaelkmelzer@gmail.com (M.K.M.); Arturo.Lopez-Martinez@lrz.tu-muenchen.de (A.L.-M.)

**Keywords:** oncolytic virus, vesicular stomatitis virus, immune-suppression, immunotherapy, vaccination, adoptive cell therapy

## Abstract

Oncolytic viruses have gained much attention in recent years, due, not only to their ability to selectively replicate in and lyse tumor cells, but to their potential to stimulate antitumor immune responses directed against the tumor. Vesicular stomatitis virus (VSV), a negative-strand RNA virus, is under intense development as an oncolytic virus due to a variety of favorable properties, including its rapid replication kinetics, inherent tumor specificity, and its potential to elicit a broad range of immunomodulatory responses to break immune tolerance in the tumor microenvironment. Based on this powerful platform, a multitude of strategies have been applied to further improve the immune-stimulating potential of VSV and synergize these responses with the direct oncolytic effect. These strategies include: 1. modification of endogenous virus genes to stimulate interferon induction; 2. virus-mediated expression of cytokines or immune-stimulatory molecules to enhance anti-tumor immune responses; 3. vaccination approaches to stimulate adaptive immune responses against a tumor antigen; 4. combination with adoptive immune cell therapy for potentially synergistic therapeutic responses. A summary of these approaches will be presented in this review.

## 1. Introduction

In recent years, great progress has been made in the development of immune-based cancer therapies in which the body’s own immune system is harnessed to fight against the invading cancer. Although immunotherapies have the potential to offer safe, systemic, and long-lasting tumor responses, the tolerogenic microenvironment of most tumors is a challenge that must be addressed in order to fully exploit the therapeutic potential of this approach. Oncolytic viruses offer a novel treatment option, due to their eloquent multimodal mechanism of action. They are probably best known for their inherent ability to cause tumor debulking via direct tumor cell lysis; however, they additionally offer the potential to break immune tolerance and stimulate potent immune responses directed against uninfected tumor cells and distant metastases. They therefore have been exploited in rationally designed combination therapies involving oncolytic viruses as immunotherapeutics. Vesicular stomatitis virus (VSV) represents a particularly attractive vector platform for viral-based immunotherapies due to its inherent tumor specificity, its rapid replication and cell-killing kinetics, its natural ability to stimulate immune responses, and the fact that there is an established genetic system available for generating recombinant vectors.

VSV is a negative-strand RNA virus from the *Rhabdoviridae* family with a relatively compact genome comprised of approximately 11,000 nucleotides encoding for five viral proteins. The VSV glycoprotein (G protein) mediates viral attachment and fusion to host cells via the ubiquitously expressed low density lipoprotein (LDL) receptor, followed by receptor-mediated endocytosis and internalization into endosomes. The low endosomal pH then triggers a conformational change in the G protein, activating fusion to the endosomal membrane and causing the release of the viral genome into the cytosol and the initiation of the replication process. The entire VSV lifecycle occurs in the cytoplasm. Despite the ability of VSV to infect a wide range of host cells, replication is limited to cells that are defective in their antiviral interferon signaling pathways, allowing for an inherent mechanism for tumor specificity. Elevated doses have been shown to result in off-target toxicities, however, and to date, only a pseudotyped VSV vaccine vector [1] and an attenuated oncolytic VSV expressing human interferon β [2] have succeeded in clinical translation, due to safety concerns of administering wild-type virus.

The hammer and anvil tactic is a military strategy that has been used since the beginning of organized warfare. This tactic involves two enemy infantry units fighting in a frontal assault, while a cavalry unit maneuvers around the enemy and attacks from behind, hammering it against the infantry line, which functions as the anvil. Generally, in order for this strategy to be successful, the force attempting the maneuver must outnumber its opponent. The concept of using a powerful oncolytic virus, such as VSV, in combination with an immunotherapeutic strategy elicits an attack against cancer in much the same way as the hammer and anvil military tactic. Although the tumor exploits various evasion and survival mechanisms, it is ultimately powerless when it is attacked from two angles, namely the direct blow from the oncolytic effect and the subsequent immune attack from behind. In this review, we will highlight the basic challenge of an immune-suppressive tumor microenvironment and then discuss a variety of strategies that have been employed using oncolytic VSV as a basis for viro-immunotherapeutics for cancer, a two-pronged approach to destroy cancer.

## 2. The Immune-Suppressive Tumor Microenvironment

Tumor development and progression is dictated by a complex interplay between tumor cells and the many components of the tumor microenvironment, including fibroblasts, extracellular matrix, blood vessels, inflammatory cells, and stimulatory molecules, such as chemokines and cytokines [3]. In order to promote their own survival, tumor cells employ a variety of mechanisms to evade the immune system and modulate the microenvironment in favor of cancer progression [4]. An important component of this process is termed “immunoediting”. This concept describes the dual role of the immune system to protect the host, as well promote tumor growth and metastases by selecting for tumor variants with reduced immunogenicity, which can thereby escape immune surveillance [5].

The development of an immunosuppressive microenvironment in tumor settings involves a multitude of players and interactions. Tumors promote immune tolerance through down-regulation of major histocompatibility complex (MHC) class I molecules and tumor-associated antigens (TAAs), thereby preventing recognition by T cells [6]. In addition, preclinical studies have indicated that tumoricidal NK cells require additional stimulatory signals, such as type I interferon (IFN) and interleukin (IL)-15, in order to exert their functions in the context of tumor-bearing hosts [7]. Tumor immune evasion is mediated, at least in part, by a network of soluble immunomodulatory factors, such as IL-6 and IL-10, as well as transforming growth factor β (TGF-β), which are secreted by tumor, stroma, and inflammatory cells [8]. These factors most likely act together to inhibit dendritic cell (DC) function and stimulate the proliferation of immune-suppressive regulatory T cells (Tregs) [9].

### 2.1. Immune Suppressor Cells

Recruitment of immune suppressor cells, such as immature DCs, Tregs, myeloid-derived suppressor cells (MDSCs), and M2-polarized macrophages function to protect the tumor from immune recognition. This is achieved through the inhibition of effector T cell proliferation, secretion of soluble immunosuppressive molecules, and obstruction of antigen presentation [10,11,12,13]. In patients suffering from melanoma, hepatocellular carcinoma, lung cancer, breast cancer and prostate cancer, the accumulation, and even the induction, of Tregs, MDSCs, and immunosuppressive tumor-associated macrophages (TAM) have been extensively characterized [14]. It was further shown in squamous cell carcinoma and basal cell carcinoma that myeloid DCs (mDCs) have diminished functions to stimulate a productive antitumor T cell response [15]. Recent evidence indicates that the degree of immune-suppressor cell infiltration in tumors correlates with overall survival or disease free survival in patients suffering from diverse cancer entities. Chevolet and colleagues have shown that there is a correlation between the numbers of monocytic and polymorphonuclear MDSCs (mMDSC and pmnMDSC), not only with reduced numbers of cytotoxic T cells, but also suppressed function [16]. Similarly, for patients with prostate cancer, higher levels of mMDSCs in the blood have been found than in healthy controls; the number of circulating DCs was reduced in these patients [17].

The immunosuppressive mechanisms of the tumor microenvironment have similarly been well characterized in a variety of mouse models. For example, mouse B16 melanoma cells have been shown to actively secrete the immunosuppressive cytokine TGF-β [18]. Raji B cell leukemias support the accumulation of antigen-specific suppressive Tregs [19]. Eisenstein et al. have shown in a metastatic Lewis lung carcinoma model in mice that MDSCs have higher tumor tropism than other immune cells including T cells, DC, cytokine-induced killer (CIK) cells, macrophages, and monocytes [20]. This was demonstrated by labeling various immune cell types with superparamagnetic iron oxide (SPIO) particles, followed by systemic injection and tracking of the labeled cells by magnetic resonance imaging (MRI) [20]. Additional lung cancer models in mice further show that there are high levels of MDSCs and M2 phenotype immune-suppressive, tumor-supporting macrophages [21]. The potential of oncolytic VSV therapy to change the phenotype of MDSCs is discussed later in this review.

### 2.2. Immune Checkpoints

Tumors can also evade immune detection by hijacking immune checkpoints, co-stimulatory or inhibitory molecules that are crucial for maintaining tolerance and modulating the duration and amplitude of immune responses in order to minimize tissue damage. Cytotoxic T-lymphocyte-associated protein 4 (CTLA-4) and programmed cell death protein 1 (PD-1) are two inhibitory receptors expressed by T cells that have become therapeutic targets to mitigate the immunosuppressive tumor environment and promote antitumor immunity. Ligation of these inhibitory molecules with their corresponding ligands on tumor cells leads to T cell dysfunction and exhaustion [22]. For example, PD-1 ligation on T cells leads to tolerance against antigens, inhibited activation, and susceptibility to apoptosis [23]. A variety of tumor entities, such as melanoma, ovarian, and lung cancer have been shown to express high levels of the PD ligand 1 (PD-L1) [24,25,26], and it is also expressed on myeloid cells of the tumor microenvironment [27]. Although it is predicted and demonstrated in several tumor models, that high levels of PD-L1 expression in the tumor correlate with poor survival in patients [28,29,30], other studies indicate that PD-L1 expression does not correlate with prognosis [31]. The contradictory data can be attributed to a variety of other factors that are associated with survival, such as cancer type, stage, and treatment history, as well as the immunohistochemistry methods employed in each study [31]. Furthermore, the outcome of immune checkpoint blockade therapy is variable amongst different tumor entities. While the most impressive responses to PD-1/PD-L1 blockade have been observed in metastatic melanoma, lung cancer, renal cell carcinoma, and hematologic malignancies [22], it has also recently been shown that triple-negative breast cancer and bladder cancer are responsive to immune checkpoint blockade [32]. On the other hand, characteristically non-immunogenic tumor entities, such as pancreatic and prostate cancers, tend to be relatively resistant to therapies with immune checkpoint inhibitors [33]. It has been speculated that co-treatment with oncolytic viral therapies could overcome tumor resistance to therapeutic immune checkpoint blockade and that these combinations could work synergistically to induce a broadened immune response compared to monotherapies [34,35]. This strategy will be discussed further in the Outlook section.

### 2.3. Dendritic Cell (DC) Maturation and Function

Thorough characterization of the tumor microenvironment has highlighted the importance of DCs as determinants of immune suppression and patient survival. Both the degree of DC infiltration, as well as the phenotype and maturation state of the cells can play a major role in modulating the local environment. There is striking evidence that plasmacytoid DCs (pDCs) are reduced in their numbers or are functionally impaired with a diminished capacity to produce type I IFNs and an enhanced ability to induce Treg expansion and induction in tumor settings [36,37,38,39]. It has been shown that the immature phenotype of DCs is mediated by tumor-induced expression of IL-10 and TGF-β. Immature myeloid DCs (mDCs) induce Treg differentiation and unresponsiveness of T cells. Furthermore, pDCs induce IL-10 production in T cells, leading to a crosstalk with mDCs and suppression of their function to prime a tumor antigen T cell response [8]. A multitude of studies have shown that both solid tumors, as well as lymphomas, have significantly decreased numbers of functionally competent, mature pDCs and an accumulation of immature and functionally impaired DCs, resulting in a reduced capacity to stimulate T cells [40,41]. In breast cancer tissue samples, it was shown that there are mostly immature DCs within the tumors, whereas mature DCs are located in the area surrounding the tumor [42]. This is in line with a similar distribution pattern observed in patients with papillary carcinoma of the thyroid, in which immature pDCs were located in the center of tumors [43]. Furthermore, in head and neck cancer patients, there is evidence that these tumors can inhibit the production of IFNα by pDCs [44]. Interestingly, the degree of pDC maturation and function has been correlated with patient survival in several cancers. A high frequency of pDCs in patients with melanoma was associated with superior survival compared to patients with low frequencies, while systemic disease was associated with significantly lower counts of pDC than in patients with non-systemic disease [28]. The overall survival of breast cancer patients was inversely correlated with accumulation of tumor-associated pDCs, while there was a positive correlation between survival and pDC concentration in blood [39]. Therefore, DCs could represent an interesting therapeutic target or predictive biomarker for immunotherapeutic approaches.

A variety of mouse models have tested the hypothesis of pDC maturation being important for antitumor immune responses. Brawand and colleagues showed that immature pDCs fail to induce antigen-specific CD4^+^ and CD8^+^ T cell responses in mice [45]. Colon carcinoma, mastocytoma, Lewis lung carcinoma and B16 melanoma models have additionally demonstrated increased percentages of immature mDCs [46]. Similarly, in mouse mammary carcinoma, there are high counts of immature pDCs, which likely support tumor growth [47]. Reduced IFNα secretion after TLR9 stimulation was shown to be a feature of pDC in a mouse model of chronic lymphocytic leukemia (CLL), as well as in human CLL patients [38]. Immature pDCs independent of tumor show impaired function to stimulate sufficient CD4^+^ T cell responses in vitro and in vivo [48]. Also in a non-tumor environment, immature pDCs were shown to correlate with a tolerogenic immune-suppression phenotype in a model of experimental glomerulonephritis in mice [49].

In summary, there are a multitude of mechanisms leading to immunosuppression in the tumor setting; not all of them can be reviewed here, as this review will concentrate on addressing VSV as an oncolytic virus, as well as a vehicle for overcoming the tolerogenic tumor microenvironment and stimulating a productive immune response directed against the tumor.

## 3. Vesicular Stomatitis Virus (VSV) as an Oncolytic Virus

Vesicular stomatitis virus (VSV) is a negative-sense single-stranded RNA Rhabdovirus, which has inherent tumor specificity and can rapidly infect and replicate efficiently in a wide range of host cells [50,51,52]. VSV replicates in the cytoplasm of susceptible cells, causing cell death as a result of the general shutdown of host RNA and protein synthesis. It was first observed that treatment of human melanoma xenografts with wild-type VSV in nude mice resulted in regression or growth inhibition of the established tumors, while sparing the surrounding normal cells [52]. We and others have since demonstrated that VSV possesses potent oncolytic properties in a variety of tumor models [53,54,55,56,57,58]. Because VSV is known to be extremely sensitive to the anti-viral actions of type I interferons (IFN), it has been postulated that its tumor specificity is the result of defects in interferon signaling in cancer cells [59]. Normal cells, which are competent in launching an efficient antiviral response quickly after infection, are able to inhibit viral replication before cell damage can be initiated. Due to compelling preclinical data demonstrating efficient tumor-specific cell lysis, two phase I clinical trials using recombinant VSV [2], have recently been initiated (ClinicalTrials.gov Identifier: NCT01628640 and NCT02923466).

## 4. Vesicular Stomatitis Virus as an Immune-Stimulating Agent

Many oncolytic viral platforms have been observed to induce antitumor immune responses as an important mechanism of action [60,61,62]. This is mediated primarily through local inflammation induced by virus infection, which stimulates the maturation of DCs and causes them to migrate to the draining lymph nodes, where they can cross-present tumor antigens to naïve T cells [63]. OV-mediated cell killing results in the release of tumor-associated antigens (TAAs), pathogen-associated molecular patterns (PAMPs) and danger-associated molecular patterns (DAMPs) from the lysed tumor cells, inducing markers of immunogenic cell death, such as membrane-associated calreticulin (ecto-CRT) and the release of high mobility group box 1 (HMGB1), ATP, and heat shock protein 70 and 90 (Hsp70 and Hsp90) [63]. Therapies utilizing VSV as an oncolytic agent have been shown to demonstrate a variety of immune responses, including the induction of tumor-specific CD8^+^ T cells that are induced following the release of tumor-associated antigens [64]. We have observed a rapid and substantial infiltration of inflammatory cells, in particular, neutrophils and natural killer (NK) cells [65,66] that are crucial for the induction of an antitumor immune response [64,67]. It has been shown in a melanoma model that oncolytic VSV induces the secretion of type III IFN IL-28 into the tumor microenvironment, causing tumor cells to display NK cell ligands and resulting in NK cell recognition, activation and cytotoxicity [67]. Important to note, however, is that this inflammatory response also represents the innate defense against the virus, and these cells are largely responsible for the rapid clearance of VSV within 72 h after treatment, having a counterproductive effect on the direct oncolytic effect [65,66,68].

An abundance of literature indicates that VSV can activate and mature pDCs, which play an important role in the detection of viral infection and in antiviral immune responses. Infection of pDC with VSV is sensed via the toll-like receptor 7 (TLR7), which mediates a maturation and activation of pDCs and can further lead to priming of CD8^+^ T cells. This maturation is characterized by the upregulation of the MHC class II molecules and CD80, CD86, CD40, which all lead to improved antigen presentation, as well as induction of high levels of type I IFNs [69,70,71]. Barchet et al. describe in detail that there is a strong induction of IFNα in pDCs after VSV infection [72]. Kawai et al. could further conclude from published data that pDCs are specialists in recognizing VSV, as opposed to their conventional counterparts, the mDCs [73]. Consistent with this, Frenz et al. showed that mDCs are more permissive than pDCs to VSV infection, and that VSV-infected mDCs were sufficient to stimulate an IFN response in pDCs without direct infection of the pDCs themselves [74]. Swiecki and his colleagues showed that pDCs are a critical component in the efficacy of VSV therapy in combination with adoptive cell therapy [75].

Oncolytic viruses are potent immunogens that activate innate antiviral immune responses that act to limit virus replication and clear the infection [65,76,77]. Despite this seemingly counterproductive effect, the innate antiviral response has the potential to mediate antitumor bystander effects through cytokine induction [67], activation of immune effector cells, such as NK cells [64,78], and through the priming of subsequent adaptive immune responses against viral and tumor-associated antigens [61,79]. It was recently shown in the B16ova model that the immune responses derived from VSV therapy are predominantly the effect of immune bystander functions resulting from the innate response to viral antigens expressed at the tumor site, even in the absence of ongoing virus replication [80,81]. This process is mediated through MyD88 signaling and innate cytokines and effector functions [64,67,77]. The role of the immune system, either as a potential inhibitor to oncolytic virus therapy or as an essential component mediating antitumor effector functions, has been intensely discussed and debated [82,83].

## 5. Strategies to Boost the Immune-Stimulating Potential of VSV

Although VSV has the inherent potential to mediate antitumor immune responses as an important aspect of oncolytic virus therapy, the prime immune effect is directed against the virus, and VSV in its wild-type form produces relatively weak immunotherapeutic responses; however, it serves as an ideal platform for the application of a variety of immune-stimulating strategies, both through viral engineering and combination approaches. These strategies will be outlined in the following sections.

### 5.1. Modification of Endogenous VSV Genes

The matrix (M) protein of VSV is responsible for many of the cytopathic effects associated with VSV infection, including the characteristic rounding of infected cells and the shutdown of host gene expression [84]. This shutdown occurs at the level of host transcription, as well nucleocytoplasmic transport of host RNA and proteins [85,86] and is responsible for the ability of VSV to inhibit activation of host IFN responses [87]. Studies have identified various mutants of VSV that possess strong IFN-inducing phenotypes, and many of these mutants harbor point mutations in their M proteins [88]. In particular, the M51R mutant was found to be defective in its ability to block host gene expression and was, therefore, able to efficiently induce IFN signaling in responsive cells [87]. Since this important finding, numerous studies have been performed using VSV-M51R or VSV-MΔ51 variants, not only in an attempt to improve safety, but also as a strategy to induce stronger virus-mediated antitumor immune responses [89,90,91].

VSV-M51R infection of CD11c^+^ mDCs in mice leads to upregulation of activation markers like CD80, CD86 and MHC class II [92]. M-mutant VSV can induce maturation of myeloid DCs (mDCs), resulting in type I IFN expression and secretion of IL-6 and IL-12 [92]. In addition, mDCs infected with rVSV-M51R effectively activate naïve T cells and show a higher capacity to induce proliferation of antigen-specific T cells and stimulate effector functions as compared to controls and lipopolysaccharide (LPS)-treated mDCs [93]. It was additionally demonstrated that VSV-MΔ51-GFP can break immune tolerance and induce long-term immune responses mediated by T cells. Lemay and his colleagues have shown that, in cells that are normally not permissive to rVSV-MΔ51 infection in vivo, a partially protective antitumor immune response, marked by maturation of DCs, can be achieved using a cell vaccine approach employing rVSV-MΔ51-GFP-infected irradiated tumor cells (B16F10 and CT26) [94].

The glycoprotein (G) of VSV is responsible for cellular attachment of the virus and subsequent entry via clathrin-mediated endocytosis [95]. Analysis of various VSV mutants harboring mutations in the G protein revealed the G6R mutant that was able to efficiently induce type I IFN responses to a greater extent than rVSV-M51R, in the absence of viral attenuation observed with the M mutant [96]. Furthermore, Janelle et al. could show that rVSV-G6R induces strong antiviral immune responses, as evidenced by production of high neutralizing antibody titers, albeit to a lesser extent than the M mutant [89]. However, the antitumor immune response induced by rVSV-M51R was more diverse and more protective than that observed for wild-type or the G6R mutant, and was mediated by a cellular immune response and characterized by a strong T cell response directed against the tumor [89]. Furthermore, it is important to consider that, in mice treated with the G6R mutant, the majority of activated T cells expressed PD-1 [89], a marker of T cell exhaustion, which could potentially play a counterproductive role in immunotherapy via PD-1/PD-L1 immune checkpoint signaling.

### 5.2. VSV-Mediated Cytokine or Immune-Stimulatory Molecule Expression

A common strategy of boosting the immune-stimulating potential of an oncolytic virus involves the incorporation of a cytokine into the viral genome. In the case of VSV, the most common engineering approach is to insert the foreign gene as an additional transcription unit, usually between the endogenous glycoprotein (G) and large polymerase (L) gene. A summary of the recombinant VSV vectors expressing immune-stimulatory cytokines/molecules that have been reported to date is provided in Table 1.

IFN-β is a member of the type I IFN family, and has both antiviral immune functions, as well as general immune-stimulatory properties. It has been hypothesized that the VSV-mediated expression of IFN-β would, therefore, not only improve the safety of VSV by restricting its replication to IFN-resistant tumor cells, but it could potentially enhance the efficacy of oncolytic VSV therapy through the induction of antitumor immune responses. In fact, mice bearing mesothelioma tumors enjoyed longer survival after local or locoregional treatment with recombinant VSV expressing mouse IFN-β than those treated with VSV expressing the human version of IFN-β, which has little cross-reactivity with mice [106]. It was shown that the improved therapeutic outcome was dependent, at least in part, on CD8^+^ T cell responses, although further studies indicated that these represented mainly a generalized T cell activation rather than tumor antigen-specific T cell effector functions [106]. Improved safety of rVSV-mIFN-β was also confirmed in this study [106]. Along similar lines, rVSV-IFN-β was shown to induce a systemic antitumor immune response, marked by increased CD8^+^ tumor-infiltrating lymphocytes (TILs) and decreased Tregs and mMDSCs, in both injected and non-injected tumors, in a model of non-small cell lung cancer [107]. Furthermore, there was evidence of the onset of a memory immune response shown via rechallenge experiments in the same study [107]. Interestingly, rVSV-IFN-β therapy was also associated with an increased expression of PD-L1 on tumor cells, both in injected and non-injected tumors, which could pose a potential limitation to the immunotherapy; however, this also indicates that combination therapies with PD-1 or PDL-1 inhibitors have the potential to result in synergistic responses. This represents an attractive strategy, since PD-1/PDL-1 inhibitors have recently entered the clinic. Based on compelling preclinical studies, a recombinant VSV vector expressing human IFN-β recently became the first VSV vector to be tested in a phase I clinical trial as an oncolytic agent, and it is being applied intratumorally to patients with sorafenib-refractory hepatocellular carcinoma (ClinicalTrials.gov Identifier: NCT01628640). A second phase I clinical trial has just recently been initiated, in which rVSV-IFNβ-NIS, additionally expressing the sodium iodide symporter (NIS), will be injected intratumorally to patients with refractory solid tumors (ClinicalTrials.gov Identifier: NCT02923466).

In a similar strategy, investigators have engineered an rVSV-MΔ51 vector encoding IFNγ [108], an important immunostimulatory and immunomodulatory cytokine that is expressed by activated NK and T cells. Mouse 4T1 mammary carcinoma and CT26 colon carcinoma tumors treated with rVSV-MΔ51-IFNγ revealed greater T cell infiltration than those receiving rVSV-MΔ51, as well as prolonged survival, which was shown to be dependent on the activation of the T cell compartment [108].

Granulocyte-macrophage colony-stimulating factor (GM-CSF) is a cytokine with strong immunostimulatory functions that induces differentiation, proliferation, and activation of macrophages and DCs, leading to cytotoxic T lymphocyte (CTL) activation [109]. In the VSV-infected cell vaccine study mentioned earlier, it was shown that vaccines consisting of rVSV-MΔ51-GM-CSF-infected cells resulted in enhanced antitumor immune responses, marked by increased numbers of activated DCs, IFN-γ-expressing NK cells, and T cells, compared to the control vector not expressing GM-CSF, even in the absence of high intratumoral replication [94]. Another group demonstrated the induction of a broad antitumor immune response by a recombinant replicating VSV (rrVSV) pseudotyped with the Sindbis virus glycoprotein and encoding mouse GM-CSF, which selectively replicates in Her2/neu-positive cells [110]. Here, Bergman and colleagues showed that the immunity is mediated by T cells, and a broad memory response was demonstrated in tumor rechallenge experiments, in which protective immune responses were also generated against tumor cells not expressing the artificially expressed human Her2/neu antigen [110].

Several interleukins have also been expressed by VSV vectors in attempts to improve immune responses. VSV recombinants expressing the suicide gene, thymidine kinase (TK), or IL-4 were shown to provide enhanced oncolytic activities compared to wild-type controls in syngeneic breast and melanoma tumors in mice, as well as increased granulocyte-infiltration and concomitant induction of antitumor cytotoxic T cell responses [50]. VSV-mediated expression of IL-12 in a murine squamous cell carcinoma model resulted in a striking reduction of tumor volume and significant survival prolongation compared to a control vector [98]. Similarly, a recombinant VSV expressing a highly secreted version of human IL-15 led to a significant enhancement of antitumoral T cell responses and prolonged survival [103]. In addition to the ability of interleukins to enhance immune responses, it was shown that VSV modified with a single chain IL-23 could provide neurologic protection, attenuating VSV replication in the central nervous system and improving the safety over wild-type VSV [100].

### 5.3. VSV as a Vaccination Platform

The concept of vaccination involves the administration of antigenic material for the purpose of stimulating the host’s immune system to develop adaptive immunity against a pathogen or disease. Therapeutic cancer vaccines aim to stimulate durable antitumor immunity, providing long-term systemic protection against relapse or metastatic disease. Effective vaccines against cancer include RNA and DNA virus platforms that deliver high concentrations of antigen to HLA class I and II molecules on DCs, activating CD4^+^ and CD8^+^ T cell responses [111]. VSV represents a particularly attractive vaccination platform due to its potent immunogenicity, leading to strong humoral and cellular immune responses [112]. Indeed, VSV-based vaccines have been shown to induce protective immunity against a variety of pathogens, including Ebola, HIV, influenza, and Marburg virus [113,114,115]. Similarly, it was hypothesized that it would be possible to enhance the therapeutic potential of oncolytic VSV therapy by incorporating a tumor-associated antigen into the vector, to generate adaptive T cell responses for protective immunity and systemic therapy against metastatic tumors [116,117].

Chicken ovalbumin (OVA) is commonly used as a model tumor antigen for cancer vaccine studies and for evaluating antitumor immune responses in preclinical studies. Recombinant VSV engineered to express the OVA antigen, injected into established B16ova tumors, was shown to efficiently prime ova-specific T cell responses compared to a VSV-GFP control vector, due to migration of VSV-OVA to the tumor-draining lymph nodes, where both viral and ova antigens could be presented, resulting in enhanced therapeutic effects [64]. However, it was subsequently demonstrated that the therapeutic effect of VSV-OVA therapy was the function of expression of a foreign, highly immunogenic antigen, whereas VSV-mediated vaccination against the self-antigen, gp100, was insufficient to improve the therapeutic outcome compared to rVSV-GFP therapy [102]. This limitation could be overcome by combination therapy with adoptive transfer of Pmel T cells specific for mouse gp100 [102], which will be discussed in greater detail in the next section. In a novel combinatorial approach, Blanchard et al. reported that stereotactic ablative radiation therapy (SABR) could control tumor growth in local, accessible oligometastatic melanoma, but produced weak T cell responses, which could be boosted by treatment with VSV expressing a tumor antigen [18], providing a potentially synergistic therapy for both local and systemic disease. In this study, it was also observed that an immune response against the self-antigen gp100 could be generated as a result of VSV-ova and SABR combination therapy, indicating a broad release of tumor antigens and subsequent antitumor immune response elicited by this therapy [18]. As an alternative to wild-type VSV as a vaccine vector, Tober and colleagues describe a novel VSV vaccine vector (VSV-GP-OVA), pseudotyped with the glycoprotein of the lymphocytic choriomeningitis virus (LCMV) [118]. This vector was just as efficient in generating OVA-specific humoral and cellular immune responses as VSV-OVA, and has the additional benefit of being the only replication-competent vaccine vector described to date that does not lose efficacy upon repeated application [118].

In contrast to the weak immune response induced by VSV-gp100, intranasal injection of a recombinant VSV expressing the human dopachrome tautomerase (hDCT) activated both CD4^+^ and CD8^+^ DCT-specific T cell responses in a murine melanoma model [117]. Bridle and colleagues then speculated that they could improve this vaccination effect by applying a prime-boost approach, which involves the expression of the same antigen by two sequentially applied heterologous viruses, in order to augment antigen-specific T cell responses. Following T cell priming with the rVSV-hDCT vector, mice bearing B16-F10 melanoma tumors were treated with recombinant adenovirus (Ad)-hDCT, which led to enhanced antitumor efficacy, and prophylactic effects were also demonstrated for this treatment regimen [117]. The same group then went on to show that VSV substantially potentiates CD8^+^ T cell responses, accelerating their progression to a central memory phenotype, when applied as a boosting vector following adenoviral vaccination [119]. They demonstrate that the application of VSV as a priming vector leads primarily to enhanced antiviral immune responses, whereas boosting with VSV elicits a stronger antitumor immune response [116]. The potency of this approach was even further enhanced by combination with a histone deacetylase (HDAC) inhibitor during the boosting phase of therapy, which functioned to inhibit the innate antiviral immune response, while enhancing the expansion of tumor-specific T cells and simultaneously depleting conventional lymphocytes and Tregs [120].

As an alternative vaccination approach, Kottke and colleagues have demonstrated that the expression of a cDNA library from a VSV vector could allow presentation of a broad repertoire of tumor-associated antigens, which reduced the emergence of treatment-resistant tumor variants [121]. Furthermore, any tumor cells that did escape the immune pressure could be treated by second-line therapy with virus-based immunotherapy [121].

### 5.4. VSV in Combination with Adoptive Cell Therapy

Adoptive cell therapy (ACT) is a highly personalized cancer therapy, in which ex vivo-expanded immune effector cells are autologously administered to the cancer-bearing host. Of the ACT approaches under investigation, adoptive transfer of T cells is perhaps the best characterized to date, with an abundance of convincing preclinical [122,123], as well as clinical [124,125,126], successes reported. Strategies to generate tumor-specific T cells for adoptive transfer include the expression of chimeric antigen receptors (CARs), which are able to recognize antigen epitopes independently of MHC presentation, or the transduction with T cell receptors (TCRs) that target a specific tumor antigen. The clinical success of CD19-CAR-engineered T cells directed against CD19^+^ B cell malignant cells has been well documented [124,125] and will likely lead to clinical approval next year by the Food and Drug Administration in the U.S. Similarly, for other tumor entities, such as melanoma, breast, colorectal, and esophageal cancers, the first evidence of positive clinical outcomes following adoptive TCR transgenic T cell therapy has been reported [126]. Despite the encouraging clinical success of these ACT approaches, toxicities in the form of cytokine release syndrome, neurotoxicity, and off-target effects have also been observed and should be carefully considered in the future clinical development of these therapies [124,125,126].

**Figure 1:**
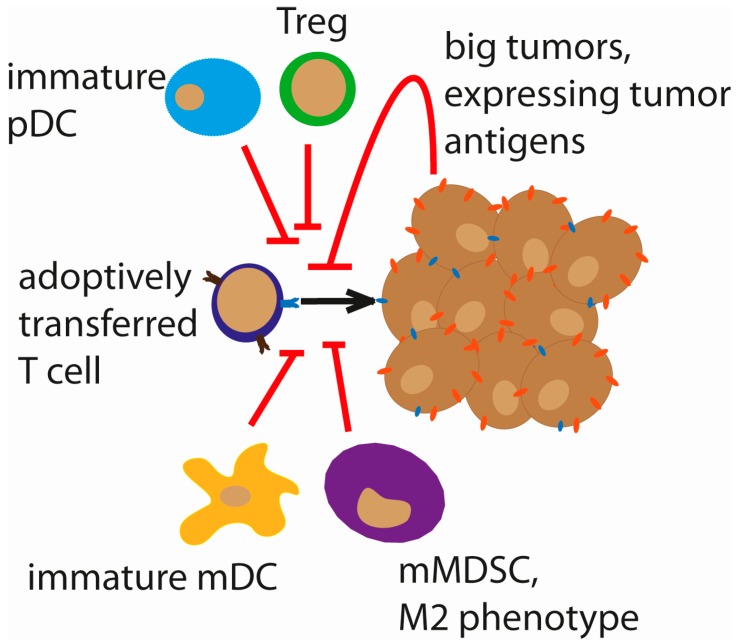
Challenges to adoptive cell therapy (ACT). The fate of adoptively transferred genetically engineered T cells (CAR/TCR) faces several hurdles. The size of the tumor can inversely correlate with the efficacy of ACT. Immature plasmacytoid dendritic cells (pDCs), myeloid DCs (mDCs), Tregs and myeloid-derived suppressor cells (MDSCs), resulting in a general immunosuppressive tumor micromilieu, are also counterproductive to ACT responses.

Although the ability of adoptively transferred antigen-specific T cells to recognize their target antigen, expand, and traffic to the tumor site has been characterized using various noninvasive imaging modalities in preclinical tumor models [127,128,129], these processes can be relatively inefficient in a more complex, immune-competent system. The immune-suppressive tumor microenvironment, reviewed extensively in the first section, also plays an important role in the fate of adoptively transferred immune cells. De Aquino et al. further characterize this immunosuppressive network, comprised of tumor-associated macrophages (TAMs), DCs, MDSCs, Tregs, inhibitory molecules (such as TGF-β, FasL, and IL-10), and immune checkpoint signals, such as CTLA4 and the PD-1/PD-1L interaction [130]. Together with the ability of tumor cells to down-regulate their MHC molecule expression to evade recognition by reactive T cells, these features all contribute to the challenges of adoptive cell therapy [130]. An interesting study by Spranger and colleagues demonstrated that, in T cell-inflamed tumors, infiltrating CD8^+^ T cells themselves can lead to Treg accumulation in the same region as the CD8^+^ T cells at the tumor site, thereby causing a suppression of the immune response [131]. Additional limitations currently faced by ACT approaches include the challenge of selecting target antigens that are truly tumor-specific and highly expressed, as well as the relative inefficiency of transferred cells to access many solid tumors [132]. Furthermore, targeting a single antigen can lead to selective pressure within the tumor and the potential for escape mechanisms leading to outgrowth of resistant tumor cells [133]. Antigenic drift has also been shown to cause alterations in epitope presentation, leading to failure of ACT, both in preclinical models [134,135] and in patients [136,137]. An overview of the major challenges to effective ACT is presented in Figure 1.

**Figure 2:**
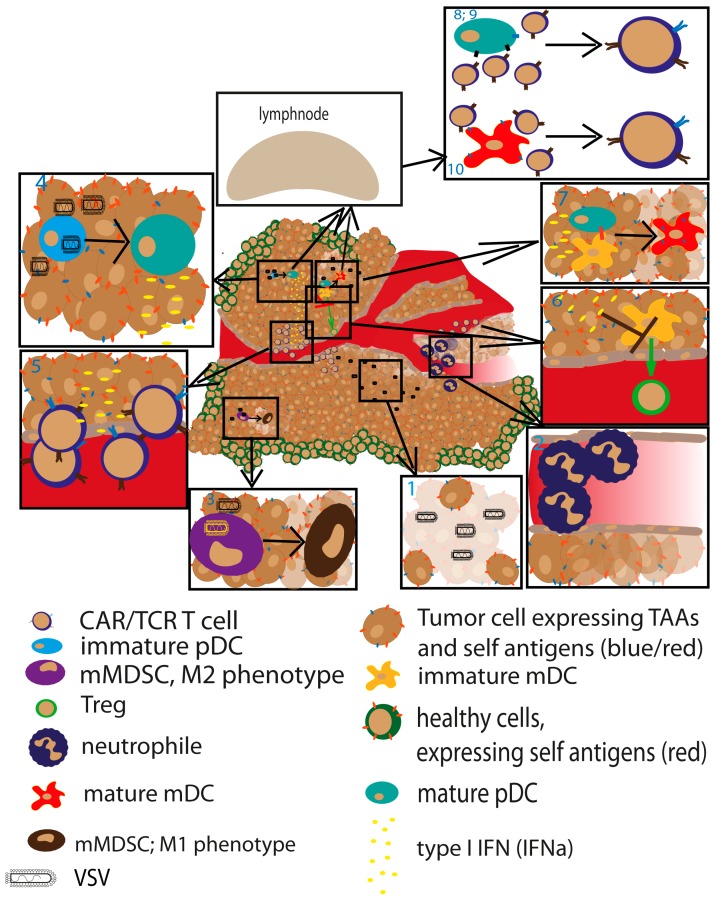
Identification of potential synergistic mechanisms mediated by ACT and VSV. Tumor debulking mediated by VSV potentially synergizes with better response in ACT. Those effects could be mediated by the following mechanisms: (**1**). VSV causes potent direct oncolysis to debulk large tumors; (**2**). Induction of necrosis by shutdown of tumor vasculature is mediated by attracted neutrophils to site of VSV inflammation; (**3**). Monocytic MDSCs (mMDSCs) are matured to a proinflammatory, tumor killing phenotype; (**4**). VSV infection causes strong induction of interferon (IFN) type I response by pDC maturation; (**5**). This IFN type I response may lead to recruitment of adoptively transferred T cells by chemokine signaling and type II IFN response of natural killer (NK) cells [141,142,143,144]; (**6**). The type I IFN response to VSV infection may lead to potentially reduced Treg attraction mediated by mDCs [145]; (**7**). TLR signaling-matured pDC can activate mDC, which is enhanced by IFNα [146,147]; (**8**,**9**). pDC prime a T cell response against viral antigens, as well against tumor antigens introduced to the viral genome [148]; (**10**). Matured mDC can take up released tumor antigens from dying tumor cells and prime a T cell response which is enhanced if there is a crosstalk between the dendritic cell subsets [148,149,150]. Black arrows within the inset boxes indicate differentiation; the green arrow indicates attraction; the T arrow represents inhibition.

The combination of ACT with oncolytic virus therapy cannot counteract all of the limitations associated with ACT monotherapies; however, there are a multitude of potential mechanisms for synergy implied by this therapeutic design. First, treatment with a potent oncolytic virus causes tumor debulking, which has the potential to enhance the efficacy of ACT. Villadangos et al. have demonstrated in an ova-expressing lymphoma model in mice, that large tumors suppress ACT function, whereas pretreatment with cyclophosphamide to shrink the tumors resulted in an enhanced therapeutic effect of the transferred OT-1 T cells [138]. We speculate that oncolytic virus therapy could mediate a similar effect. Secondly, while ACT is dependent on the adequate expression of the targeted tumor epitope, oncolytic virus therapy has the potential to infect and kill all tumor cells, indicating that this combination could compensate for the challenge of antigen loss, drift, and shift associated with ACT targeting a single antigen. Finally, we hypothesize that oncolytic virus therapy can mitigate the suppressive activities of MDSCs and Tregs, which have been shown to inhibit ACT [19,139,140], via their known capacity to modulate the MDSC phenotype to a more proinflammatory and cytotoxic one [20] and to activate T cells through DC maturation and IFN-α secretion [69,72]. These interactions between oncolytic virus therapy and ACT are mainly speculative based on the currently available literature, however, and additional studies aimed at investigating the interplay between virus-infected cells and DC maturation and T cell activation and recruitment are needed in order to fully elucidate the potential synergism of the combination therapy. Because this review focuses specifically on VSV, we will now summarize the most relevant literature describing the combination of VSV with ACT. A summary of the interplay between oncolytic VSV immunotherapy and ACT is depicted in Figure 2.

Although combination therapies employing oncolytic VSV and adoptive cell therapies are still in their preliminary stages, with not much literature yet available, the majority of these reports involve the adoptive transfer of T cells, as opposed to other immune cell types. Wongthida et al. have reported that intratumoral application of rVSV-ova in a subcutaneous B16ova melanoma model significantly enhances the activation level of adoptively transferred naïve OT-I T cells against the ova peptide, an effect that was mediated by CD11c^+^ DC [102]. These findings corroborated previous reports from Diaz and colleagues, in which it was shown that even rVSV-GFP, which does not express the ova antigen, could cause enhanced activation of naïve OT-I T cells in comparison to monotherapy with either VSV or the T cells alone, although to a lesser extent than that observed when rVSV-ova and OT-I T cells were applied [64]. As a follow-up to these studies, the authors investigated vaccination with rVSV expressing the self-antigen gp100, which provided no additional therapeutic effects over rVSV-GFP, but could mediate an enrichment of antigen-specific T cells and impressive cure rates when B16ova-bearing mice were pre-treated with naïve Pmel T cell transfer [102]. In contrast, the transfer of naïve Pmel T cells alone was not sufficient to eradicate tumors, highlighting the potential benefit of combining adoptive T cell therapy with oncolytic VSV [102]. To further improve the combination therapy, Rommelfanger et al. used a combination of systemic rVSV-ova, rVSV-hgp100, and the transfer of naïve OT-I T cells and Pmel T cells in this B16ova melanoma model [151]. They demonstrated that by simultaneously targeting two distinct tumor antigens, they could achieve regression of all treated tumors, and long-term, tumor-free survival [151]. In this study, the authors also noted that the major therapeutic mechanism appeared to be the priming of an antitumor immune response, rather than the adoptive cell therapy. This was evidenced by the combination of rVSV-hgp100 with Pmel T cells increasing the prevalence of pDCs in the tumor-draining lymph nodes and spleens of those treated mice, which could then present antigen to the increased pool of naïve T cells provided by the adoptive transfer [151]. This combination leads to the more rapid onset of an immune response against the tumor antigen than that which can be achieved without increasing the number of antigen-specific naïve T cells before priming [151]. In subsequent work with the B16ova melanoma model, this group could observe efficient priming of OT-I T cells against the ova-bearing tumors if combined with rVSV-ova [18]. In another model, Gao and colleagues have demonstrated that viral infection of meningeal tumors with rrVSV could break the blood-meningeal barrier and allow efficient infiltration and proliferation of adoptively transferred memory T cells [152]. In this study, it was shown that viral infection shifted the tumor and meningeal infiltration of leukocytes from consisting mainly of macrophages to predominantly T cells [152]. They further went on to show that therapy of peritoneal breast tumor metastases generates memory T cells that prevent the establishment of meningeal tumors in the same animals [152].

Another benefit of combining oncolytic virus therapy with ACT is the potential to exploit the adoptively transferred cells as carriers to shield the virus from inactivation and nonspecific uptake and delivering it to the tumor target via the intrinsic homing mechanism of the cells. Qiao et al. showed that rVSV-MΔ51 replicated poorly in OT-I T cells, but enhanced their effector functions in vitro and resulted in higher rates of tumor homing in vivo, compared to uninfected OT-I cells [153]. Furthermore, induction of endogenous antitumor T cell responses were observed, and this combination resulted in superior therapeutic effects in vivo compared to virus or T cells alone in the B16ova tumor model [153]. Loading with rVSV-GFP was also sufficient to improve the therapeutic effects of OT-I T cells in the same tumor model, and this therapy could be even further enhanced through Treg depletion and administration of IL-2, which provided multiple therapeutic mechanisms, including improved OT-I T cell persistence in vivo, enhanced VSV delivery to tumors, and activation of NK cells [154]. An additional benefit of this therapy was the protection of VSV from neutralizing antibodies when low multiplicities of infection (MOI) of VSV were loaded onto OT-I T cells [154]. In an alternate study using rVSV-GFP, it was shown that loading VSV onto antigen nonspecific T cells was sufficient to clear metastases in immune-competent mice bearing metastatic B16ova tumors [155]. While naked VSV failed to elicit a sufficient antitumor immune response in this model, when it was loaded onto T cells, induction of tumor-reactive lymphocytes against the self-antigen Trp-2 was observed [155]. Partial remissions in mice could also be achieved in LLC-ova (Lewis lung carcinoma) and CMT93tk (colorectal carcinoma) tumor models using lymphocytes loaded with VSV, while the response was considerably weaker in mice treated with either VSV or T cells alone [155]. Similar to OT-I T cells, CAR T cells directed against the Her2 antigen did not lose their function or viability upon infection with rVSV-MΔ51-GFP, and they were shown to maintain their antitumor effector functions and efficiently deliver virus to the tumor [156]. The synergistic effect of the combination therapy was further highlighted in vitro, where it was shown that the D2F2/E2 cell line, which is rather resistant to VSV or CAR T therapy alone, could be efficiently killed by the combination [156].

Although the majority of reported VSV and ACT combination therapies involve the use of T cells, dendritic cells also represent good candidates for adoptive cell transfer. DC-based vaccines represent a promising immunotherapeutic strategy due to their ability to activate both innate immune effectors, including NK cells, as well as antigen-specific T cell immunity. A study by Boudreau et al. demonstrated that infection of CD11c^+^ DCs with an rVSV-MΔ51 vector caused DC activation, marked by production of proinflammatory cytokines (IL-12, TNF-α, IFN-α/β) [99]. Adoptive transfer of DCs infected with rVSV-MΔ51 expressing the ova-derived SIINFEKL epitope to mice bearing lung metastases of melanoma mediated substantial tumor control via engagement of NK and CD8^+^ T cells, an effect that was completely abrogated by depletion of NK cells [99].

## 6. Summary and Outlook

In this review, we have provided a broad overview of the use of vesicular stomatitis virus (VSV) as a potent oncolytic agent and a mediator of immunotherapeutic antitumor effects. We have described in detail the immunosuppressive microenvironment characteristic of tumors, which presents a major challenge to immune-based approaches for treating cancer and poses numerous arguments for the application of VSV as an ideal platform for overcoming this hurdle. We further summarize a variety of strategies that have been applied to increase the inherent immune-stimulating potential of VSV, including virus engineering, vaccination approaches, and combination with adoptive cell transfer.

Despite the compelling evidence presented here for the use of VSV as a viro-immunotherapeutic, acting both as a hammer and anvil to destroy tumors from multiple angles, the unfortunate truth is that VSV in its wild-type form is neurotropic and can cause dose-limiting encephalitis, severely restricting the clinical translation of this vector platform. Although an oncolytic VSV has made its way to phase I clinical trial, it is attenuated via viral-mediated interferon (IFN)β expression, which has been shown to improve the safety of the virus. Additional strategies to improve the safety of VSV include pseudotyping with the glycoprotein of a heterologous virus to alter the tropism. To this end, rVSV-GP, which is pseudotyped with the glycoprotein of lymphocytic choriomeningitis virus, has been shown to be not only safe, but an efficient vaccination vector [118]. We speculate that safer engineered VSV vectors such as these will be instrumental in moving VSV forward in clinical application.

Furthermore, it is important to note that many of the studies presented here were conducted in overly simplified preclinical models which often poorly reflect the clinical scenario. In order to better predict the efficacy of these therapies upon clinical translation, the application of new treatment strategies to genetic models of cancer are warranted. Furthermore, many of the investigations presented here utilize model tumor antigen systems (such as the B16ova/OT-I model) as the platform for testing immune-based therapies. Although these are important models for proof-of-principle studies, it will be of utmost importance to carry out follow-up studies targeting real tumor antigens expressed at physiological levels to recapitulate the scenario that would be encountered in a clinical tumor setting.

Finally, in an era where immune checkpoint inhibitors are taking center stage as the next generation of cancer therapeutics, it is only logical that their combination with oncolytic virus therapy would be investigated. Blockade of immune checkpoints to release the immune-suppressive tumor microenvironment, in combination with the immune-stimulating effect of an oncolytic virus has the potential to provide an extremely effective therapeutic approach. In fact, the first reports of such combinations have already been published, and it is expected that such investigations will become more prominent in the coming years. Preliminary findings indicate that the interactions between the immune checkpoint inhibitors and the oncolytic virus are quite complex, and the optimal selection of viral vector and immune checkpoint to be inhibited, as well as the timing of administration of the two therapeutic agents, are all crucial to the ultimate success of the therapy [157]. Combination of recombinant replicating VSV (rrVSV) expressing granulocyte-macrophage colony-stimulating factor (GM-CSF) with an anti-CTLA4 (cytotoxic T-lymphocyte-associated protein 4) monoclonal antibody was successful in eliminating implanted tumors, as a function of CD4^+^ and CD8^+^ T cell responses [158]. In a similar study, a VSV vector encoding for mouse IFNβ and the sodium-iodide symporter (rVSV-mIFNβ-NIS) was applied in combination with anti-PD-L1, which resulted in the enhanced therapeutic outcome of the oncolytic virus therapy by application of immune checkpoint blockade in mice bearing acute myeloid leukemia (AML) [159]. As the potential interactions between the different modalities of immunotherapeutics become better elucidated, it is expected that new, rationally designed combination therapies with oncolytic virus vaccines, adoptive cell transfer, and immune checkpoint inhibition will change the face of cancer therapeutics and provide urgently needed alternatives for aggressive metastatic disease.

## Figures and Tables

**Table 1:** Preclinical and clinical recombinant vesicular stomatitis virus (VSV) vectors.

Virus	Modification	Action	Murine Tumor Model	Reference
VSV IL-4	Expression of IL-4 Expression of TK	Oncolytic Immunogenic	Melanoma Mammary Adenocarcinoma	[50]
VSV mIFNβ VSV hIFNβ VSV rIFNβ	Expression of IFNβ gene murine (m), human (h), rat (r)	Oncolytic Immunogenic	Mammary Adenocarcinoma	[97]
VSV IL-12	Expression of IL-12	Oncolytic Immunogenic	Squamous Cell Carcinoma	[98]
VSV ova	VSV expression of chicken ovalbumin	Oncolytic Immunogenic	Melanoma	[64]
VSV hDCT	VSVmΔ51 expression of human DCT	Oncolytic Immunogenic	Melanoma	[99]
VSV IL-23	Expression of IL-23	Oncolytic Immunogenic Attenuation in the CNS	Mammary Adenocarcinoma	[100]
VSV IL-28	Expression of IL-28	Oncolytic Immunogenic	Melanoma	[67]
VSV Flt3L	VSVmΔ51 expression of human Fl3L (growth factor DC’s activator)	Oncolytic Immunogenic	Lymphoma Melanoma	[101]
VSV hgp 100	VSV expression of hgp100 a tumor-associated antigen	Oncolytic Immunogenic	Melanoma	[102]
VSV IL-15	VSVmΔ51 expression of IL-15	Oncolytic Immunogenic Safer	Colon Adenocarcinoma	[103]
VSV H/F, VSV aEGFR VSV aFR VSV aPSMA	VSV Pseudotyped lacking G gene Displaying single chain antibodies (ScFy)	Oncolytic Immunogenic	Myeloma	[104]
VSV HIV-1 gp 160	VSV expression of human immunodeficiency virus 1 Hybrid fusion protein 160 G	Oncolytic Immunogenic	Leukemia	[105]
**Virus**	**Modification**	**Action**	**Clinical Trial**	**Reference**
VSV rIFNβ	Expression of IFNβ gene	Oncolytic Immunogenic	Phase I Hepatocellular carcinoma	NCT01628640
VSV IFNβ-NIS	Expression of the sodium iodine symporter (NIS) and human interferon β (IFNβ)	Oncolytic Immunogenic	Phase I Refractory solid tumors	NCT02923466
